# Dietary patterns and psoriasis severity in Thai patients: a machine learning approach for small sample data

**DOI:** 10.1038/s41598-025-17657-z

**Published:** 2025-09-26

**Authors:** Pichit Boonkrong, Subij Shakya, Wantika Kraunamkam, Teerawat Simmachan

**Affiliations:** 1 College of Biomedical Engineering, Rangsit University, Pathum Thani, 12000 Thailand; 2 Department of Food and Nutrition, University of Helsinki, 00790 Helsinki, Finland; 3 Department of Medical Sciences, Faculty of Science, Rangsit University, Pathum Thani, 12000 Thailand; 4 Thammasat University Research Unit in Statistical Theory & Applications, Pathum Thani, 12120 Thailand

**Keywords:** Chronic disease, Dietary habits, Feature selection, High dimensionality, Tom Yum Kung, Health care, Medical research, Risk factors, Signs and symptoms, Mathematics and computing

## Abstract

This study investigates the relationship between dietary patterns and psoriasis severity using advanced machine learning (ML) techniques. The dataset, comprising 37 features including demographic, clinical and dietary features from 142 Thai psoriasis patients, exhibits moderately high dimensionality typical of clinical studies. To address limitations posed by the small sample size, a hybrid resampling strategy integrating bootstrapping with *K*-fold Cross-Validation (CV) was implemented. Using Random Forest (RF) and eXtreme Gradient Boosting (XGB), a total of 60 classification models were evaluated by varying train/test splits and applying multiple feature selection methods, including Least Absolute Shrinkage and Selection Operator (LASSO), Mean Decrease Accuracy (MDA), and Mean Decrease Impurity (MDI). Although bootstrapping alone sometimes resulted in overfitting, its combination with *K*-fold CV improved generalizability. In optimal configurations, both RF and XGB achieved sensitivity, specificity, and F1-scores exceeding 90%, alongside area under the curve (AUC) values above 95%. SHapley Additive exPlanations (SHAP) analysis revealed key dietary factors associated with increased psoriasis severity, including high-sodium foods, processed meats, alcohol, red meats, fermented products, and dark-colored vegetables. Clinically, prioritizing weight management is essential, as Body Mass Index (BMI) arose as the strongest feature of psoriasis severity. Dietary triggers identified in this study should inform comprehensive care plans. Popular Thai cuisines, especially *Tom Yum Kung* emerged as a potentially suitable option, while *Som Tum*, *Pad Thai*, *Moo Kratha*, and *Khao Niao Mamuang* were identified as potential triggers when consumed excessively. These findings highlight the importance of dietary moderation and personalized guidance, supporting health literacy, patient management, and smart healthcare innovations in Thailand.

## Introduction

**Fig. 1:**
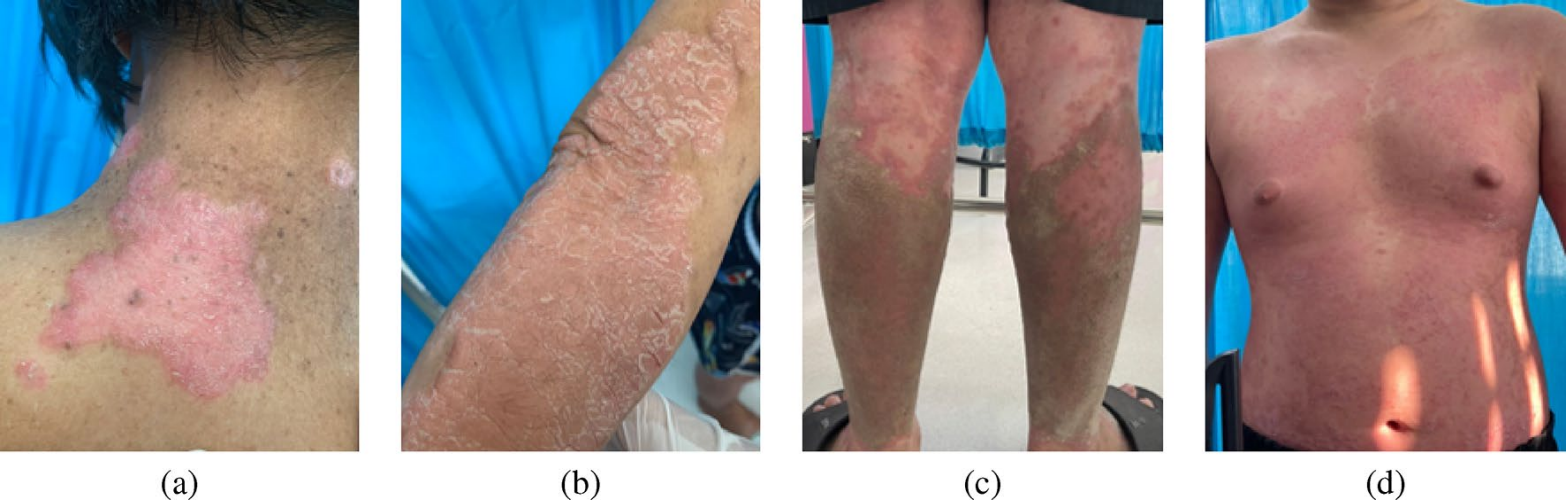
Clinical presentation of plaque psoriasis in the study cohort. The images show characteristic well-demarcated, erythematous plaques with overlying silvery scales at various anatomical locations: **a** Cervical region, **b** Posterior forearm, **c** Lower extremities, and **d** Thorax.

Psoriasis is a chronic inflammatory skin condition characterized by abnormal T-helper 17 cell immunity and keratinocyte dysfunction, leading to thick, red patches covered with silvery scales^1,2^. Living with psoriasis poses significant challenges, particularly in moderate to severe cases, as illustrated in Fig. 1, where lesions on the neck, hands, legs, and torso not only cause physical discomfort but also interfere with mobility, daily tasks, and social interactions^3–5^. While the precise etiology remains unclear, multiple studies have suggested that nutrition plays a significant role in psoriasis development and its associated comorbidities^6–10^. Interestingly, the identification of specific dietary components or patterns within Thai cuisine that modulate psoriatic inflammation in Thai patients could provide the empirical rationale for developing targeted nutritional interventions aimed at augmenting standard therapeutic regimens and improving their quality of life (QoL).

Recent researches have explored the established influence of other personal and lifestyle factors on the disease’s clinical presentation^4–6,9–12^. Machine Learning (ML), a subset of Artificial Intelligence (AI), enables computers to learn from data patterns, with key applications in dermatology^13–17^. ML has expanded to psoriasis research, analyzing disease progression, treatment efficacy, and patient outcomes^17–21^. Furthermore, ML approaches aid in psoriasis diagnosis, severity assessment, and treatment prediction and management. Studies identified Tumor Necrosis Factor (TNF)-regulated genes as potential biomarkers for treatment response^18^ and developed predictive models forecasting 12-week psoriasis outcomes, offering a non-invasive alternative to biopsies^19^. Classifiers using dermal biomarkers and clinical data improves drug response predictions, advancing precision medicine and reducing healthcare costs^20^. Digital health platforms, AI-powered nutritional guidance, and telemedicine are increasingly recognized for their role in enhancing patient engagement and optimizing psoriasis management^13,14,16,17,22^. Notably, AI-based systems such as the *SkinTeller App* on *WeChat* have demonstrated superior accuracy to dermatologists in estimating Psoriasis Area and Severity Index (PASI), thereby improving both clinical assessments and patient self-management^17,22^. Additionally, computer-aided diagnostic tools incorporating ensemble convolutional neural networks can detect and classify seven distinct psoriatic subtypes, while offering treatment recommendations through multi-criteria decision-making frameworks^21^.

In Thailand, unique dietary patterns incorporating a variety of seafood, fermented foods, tropical fruits, and high-sodium condiments differentiate local eating habits from those in other regions. Despite increasing interest in the potential impact of dietary intake on psoriasis severity, the relationship remains largely under explored, necessitating more sophisticated analytical techniques such as ML. Given the multifaceted nature of dietary intake and its effects on psoriasis, advanced ML models were employed to identify key dietary contributors to disease severity and to provide actionable insights for patient management. However, ML models typically require large sample sizes to generalize effectively, particularly when handling high-dimensional data. A general rule of thumb suggests that the number of observations (*n*) should be at least 10 times the number of features (*m*) to ensure robust model performance^23,24^. In clinical research, when the number of features is high relative to the sample size, models tend to suffer from overfitting, leading to inflated performance on training data but poor generalization to unseen cases. With 37 features and only 142 Thai psoriasis patients in this study, the event-per-predictor (EPP) ratio is $n/m = 142/37 \approx 4$, far below the recommended threshold, indicating a heightened risk of model instability and overfitting.

To address the limitations of the dataset, a hybrid resampling strategy integrating bootstrapping and *K*-fold Cross Validation (CV) was employed. This approach was designed to stabilize model performance while ensuring a robust evaluation framework. Three feature selection techniques including Least Absolute Shrinkage and Selection Operator (LASSO), Mean Decrease in Accuracy (MDA), and Mean Decrease in Impurity (MDI) were applied to identify the most informative features. Subsequently, a total of 60 classification scenarios were experimented by training two classifiers, Random Forest (RF) and eXtreme Gradient Boosting (XGB), on various feature sets and resampling conditions. Finally, SHapley Additive exPlanations (SHAP) was utilized on the best-performing model to identify and interpret the key dietary contributors to psoriasis severity. The resulting framework is intended to provide clinically meaningful insights and guide personalized dietary recommendations for psoriasis management in Thailand.

## Machine learning framework

**Fig. 2:**
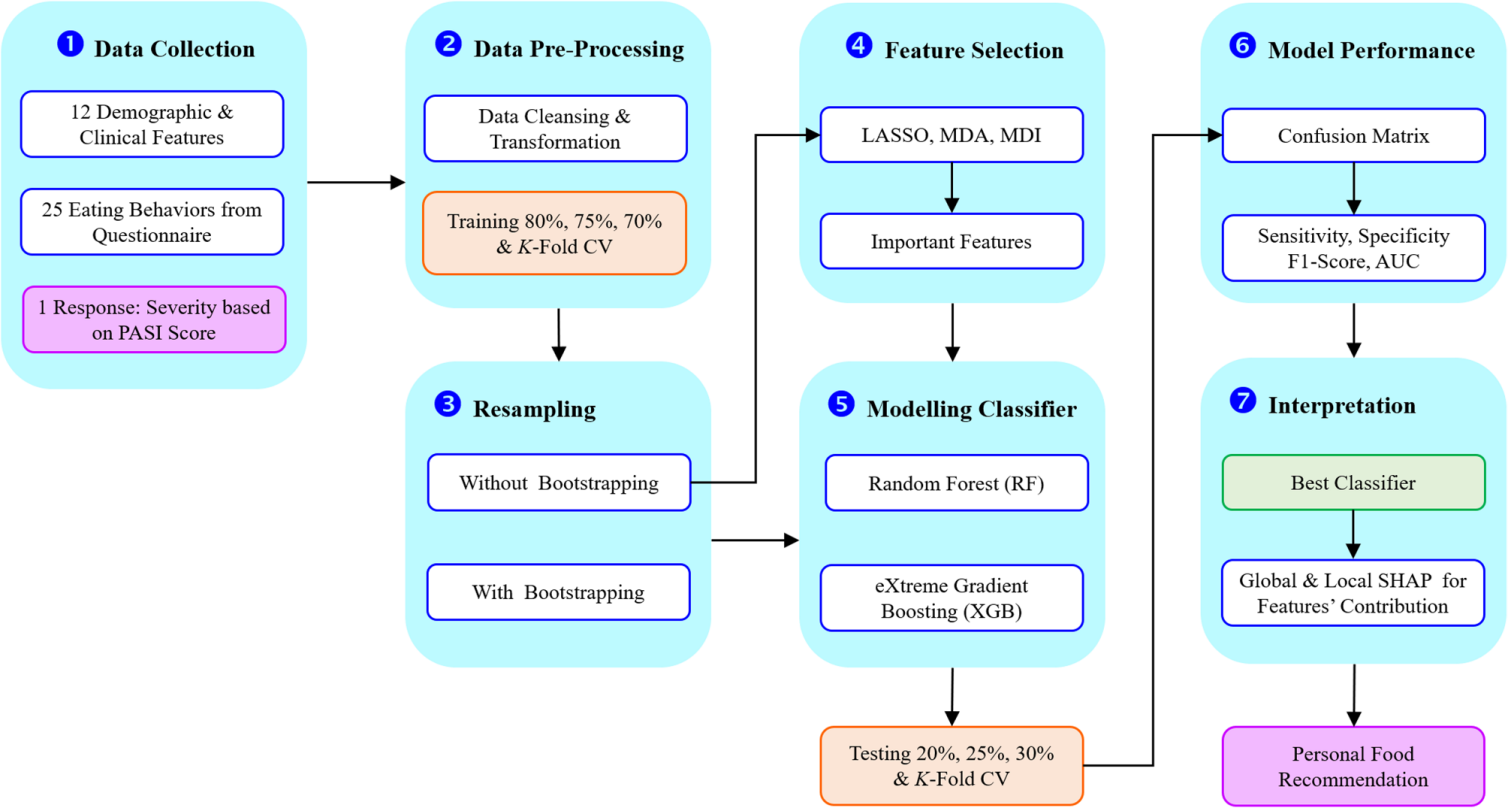
The seven-stage classification pipeline is initiated by data collection, and is followed by pre-processing. Resampling is performed with and without bootstrapping, then feature selection is applied using LASSO, mean decrease accuracy (MDA) and mean decrease impurity (MDI). Random Forest and eXtreme Gradient Boosting models are trained and evaluated via confusion matrix, sensitivity, specificity, F1-score and AUC. Finally, global and local SHAP analyses interpret the best classifier’s feature contributions to inform personalized food recommendations.

Focusing on the potential relationship between food consumption and psoriasis severity, the cutting-edge ML methodologies were the main research tools. Due to patient recruitment difficulties, high data acquisition costs, and the intrinsic rarity of certain diseases, clinical datasets are often small, posing a significant bottleneck for training robust and reliable ML models. Motivation and problem specification are firstly illustrated. Classifying psoriasis severity, the methodical workflow is visually exhibited in Fig. 2. The detailed breakdown is as follows:

### Motivation and problem specification

Thailand’s dietary habits, characterized by a variety of foods, differ from those in other regions. While interest in the impact of diet on the psoriasis severity is increasing, research in this area remains limited. Thus, it is interesting to develop an ML framework to classify psoriasis severity and identify significant dietary features. The dataset, $D = \{(X_j, Y_j) \mid j=1, \dots , n\}$, was collected from $n=142$ psoriasis patients. It comprises $m=37$ features ($X \in \mathbb {R}^m$) and a binary response variable ($Y \in \{0, 1\}$) indicating low or moderate-to-high severity. Given the low EPP ratio ($n/m < 10$), bootstrapping was employed to generate *B* resampled datasets, $D^{*(b)}$, to enhance model stability. A *K*-fold CV scheme was then applied for robust performance evaluation. The framework employs a multi-stage feature selection and classification process. First, LASSO logistic regression was implemented to select an initial subset of predictive features by varying a tuning parameter $\lambda$. These features were then ranked using two RF-based techniques: MDA and MDI scoring. Subsequently, RF and XGB classifiers were trained on the three distinct feature sets derived from LASSO, MDA, and MDI. The performance of these models, denoted as $\hat{Y} = f(X; \theta )$, was evaluated using sensitivity, specificity, F1-score, and Area Under the Curve (AUC) to identify the optimal model and feature set. Finally, SHAP values were calculated for the best-performing model to provide interpretable insights into individual feature contributions.

### Data collection and description

The study protocol was approved by the relevant institutional ethics committees, and all 142 participants provided written informed consent in accordance with the Declaration of Helsinki. The questionnaire was designed into two main parts (see Supplementary Material 1). In Part I, 12 features were collected, including two numeric (age, BMI) and 10 categorical variables covering demographics, health features, and lifestyle. The response variable, psoriasis severity, was categorized as binary (low vs. moderate-to-high). In Part II, dietary patterns were assessed using a 25-item food frequency questionnaire focused on Thai cuisine. These items were treated as 25 additional variables, with consumption frequency measured on a 4-point scale: never (0), 1-2 times/two weeks (1), 1-2 times/week (2), and $\ge$3 times/week (3). Descriptive statistics for the dataset, including demographic, clinical, and dietary information, are presented in Tables S1 and S2.

### Data pre-processing

Data pre-processing is a critical step in addressing the challenges of moderately high-dimensionality in this study. It includes data cleansing, transformation, splitting training/testing sets, bootstrapping, and *K*-fold CV. These steps ensure data quality, enhance model performance, and enable reliable evaluation while mitigating the risks of overfitting and instability in predictive modeling.

#### Data cleansing and transformation

Initially, missing data were identified and removed, reducing the sample size from $n = 159$ to $n = 142$ complete records. The response variable *Y*, derived from the PASI score, was encoded as binary, i.e., $Y = 0$ for $\text {PASI} < 10$ (low severity), $Y=1$ for $\text {PASI} \ge 10$ (moderate-to-high severity). The features included 12 basic demographics and 25 attributes of the dietary styles. All 10 categorical demographic variables were transformed into numeric format using dummy encoding. The 25 dietary pattern features, along with age and BMI, were treated as numeric and normalized using *Z*-score scaling to minimize noise and ensure comparability.

#### Training and testing sets

To ensure robust model evaluation and address class imbalance, the dataset ($n=142$) was partitioned using stratified random sampling. Three train/test split ratios (70/30, 75/25, and 80/20) were evaluated, with specific instance counts for each scheme detailed in Table S3. To counteract class imbalance, oversampling was applied exclusively to the training sets. The impact of resampling was further assessed by evaluating both RF and XGB classifiers with and without bootstrapping. This methodology, combined with *K*-fold CV, was designed to provide a stable and reliable performance assessment.

#### Bootstrapping

With the limited sample size causing difficulty in finding the final model, it was challenging to figure out the significant features. Calculating the EPP ratio, $142/37=3.8378$ indicating insufficient requirement for developing predictive model^23–25^. Bootstrapping was applied to address the small sample size by generating resampled datasets from *D* through sampling with replacement. Each resampled dataset $D^*{(b)} = \{(X_j, Y_j) \mid j \in S^{(b)}\}$, where $S^{(b)} \subseteq \{1, \dots , N\}$, contained $n_b = 75$ samples. Five rounds of bootstrapping were conducted, producing an augmented training set $n_\text {train} = 5 \times n_b = 375$. The bootstrapping process generated augmented training sets by resampling the original training data with replacement while maintaining the same testing set for each split ratio. For each bootstrap iteration, multiple resampled datasets were created, preserving class proportions. As shown in Table S3, the training sets of standard ratios were initially included and the proportions of classes $Y=0$ and $Y=1$ were maintained as 209/375 and 166/375 as their original proportion were 79/142 and 63/142, respectively. Importantly, the testing set remained independent to ensure unbiased evaluation.

#### *K*-fold cross validation

The $K$-fold CV was employed to evaluate model performance reliably. The dataset $D$ was partitioned into $K$ subsets, $D_1, D_2, \dots , D_K$, of approximately equal size. For each fold $k$, the training set was $D_\text {train}^{(k)} = \bigcup _{j=1, j \ne k}^K D_j,$ and the test set was $D_\text {test}^{(k)} = D_k$. This process, repeated for $K = 5$ and $K = 10$, ensured all data points were used for training and testing. By averaging results across folds, $K$-fold CV provided robust and unbiased performance estimates. Prior to adopting the $K$-fold CV process, stratified random sampling was used to ensure approximately balanced class representation while utilizing the full dataset for training and validation. For the original $K$-fold CV ($N = 142$) as shown in Table S3, the dataset was split into $K$ folds while maintaining class proportions. In 5-fold CV, each fold contained approximately $28-29$ instances of $Y=0$ and $12-13$ of $Y=1$. For 10-fold CV, each fold included $14-15$ instances of $Y=0$ and $6-7$ of $Y=1$.

#### Hybrid resampling

To further enhance classification performance, a hybrid approach integrating bootstrapping and $K$-fold CV was implemented. Bootstrapping increased the training set size, while *K*-fold CV partitioned the augmented dataset into folds for robust evaluation. To maintain a consistent training set size, 5-fold and 10-fold CV were implemented. As shown in Table S3, for 5-fold CV, each fold contains approximately $28-29$ instances of $Y=0$ and $15-16$ of $Y=1$ in the training set, with testing sets comprising $12-13$ for $Y=0$ and $12-13$ for $Y=1$. For 10-fold CV, each fold reduces the training size per iteration, with approximately $14-15$ for $Y=0$ and $7-8$ for $Y=1$ in the training set, while testing sets comprise $6-7$ for $Y=0$ and $6-7$ for $Y=1$. Stratified sampling ensured proportional class representation in each fold, preserving data integrity. The hybrid resampling technique mitigated the limitations of a small sample size, allowing models to learn generalizable patterns while providing reliable performance metrics.

### Feature selection

Maximizing the accuracy of the classification, a subset of features from the original set was selected by the feature selection process. Identifying and prioritizing the most influential features from the available 37 featuress, feature selection techniques including LASSO, MDA, and MDI can significantly enhance psoriasis severity classification models.

#### LASSO technique

LASSO regularizes the model by adding a penalty term based on the *L*1 norm of the coefficients^26–29^. This shrinking effect drives some coefficients to zero, effectively excluding them from the model and achieving crucial features. Given a set of features, $X_1,X_2,..., X_{m}$, binary logistic regression is employed to model a dichotomous response variable. The model utilizes a logit link function to relate the probability of an outcome to a linear combination of predictor variables. The specific model described extends the standard framework by incorporating penalty term with *L*1 regularization, $P_{\lambda }(\varvec{\beta })$, whose magnitude is controlled by a tuning parameter $\lambda \ge 0$. Instead of maximizing the log-likelihood function, the coefficient estimates, $\hat{\beta }$, are derived by minimizing the negative log-likelihood function combined with this penalty term. The choice of $\lambda$ is crucial as it balances the model’s variance and bias, with CV being a common selection method. The resulting LASSO estimates, $\hat{\beta }$, revert to the traditional maximum likelihood estimates (MLE) when $\lambda = 0$. For this study, $\lambda$ values were evaluated in the range of $10^{-3}$ to $10^3$. Subsequently, an optimal $\log (\lambda )$ of approximately $-2$ was obtained to minimize misclassification error and its plot is given in Fig. S1. Following this, MDA and MDI were used to evaluate feature importance in RF and XGB models built upon these LASSO-selected features.

#### Mean decrease accuracy and impurity

MDA and MDI quantifies a feature’s contribution to RF model accuracy^25,30^. Higher MDA or MDI scores indicate greater predictive importance. Let $S_B$, $S'_B(X_i)$, $A_0$, $A'(X_i)$, and $\Delta A (X_i)$ denote out-of-bag samples, permuted out-of-bag samples, baseline accuracy, permuted accuracy, and accuracy decrease for feature $X_i$, respectively. The MDA scoring procedure follows the algorithm S1. On the other hand, MDI measures a feature’s role in reducing impurity at decision nodes in RF. Higher MDI scores indicate greater importance in distinguishing response variable classes. Let $p_i$, $c$, $n$, $T$, $N \in t$, $n_L$, and $n_R$ denote the proportion of samples belonging to class $i$ at node $N$, the number of classes, the number of samples at node $N$, the total number of trees, the number of nodes in tree $t$, and the number of samples in the left and right child nodes, respectively. The algorithm S2 outlines the MDI scoring process.

### ML classifiers and evaluation metrics

ML classifiers have gained prominence to support medical diagnostics, particularly for complex conditions like psoriasis. This study explores the application of two powerful ML algorithms, RF and XGB, in predicting psoriasis severity using high-dimensional data from small sample sizes.

#### Random forest

RF was initially introduced by Breiman and it is a powerful ensemble technique combining predictions from multiple decision trees^31^. Each tree is built using bootstrap samples and certain numbers of features (*k*). At each node in each bootstrap sample, $k \approx \sqrt{m}$ features were randomly selected from total number of features (*m*) based on feature splitting criteria or feature importance scores such as MDA and MDI^30,32^. The final prediction is made by a majority vote (for classification tasks) amongst the trees, leading to improved accuracy and robustness^32–34^. Classifying psoriasis severity, the RF classifier has been applied directly and indirectly as hybrid ML.

#### eXtreme gradient boosting

XGB is an advanced ensemble learning algorithm that builds a strong predictive model by combining multiple weak learners, typically decision trees^30,35^. It employs a sequential boosting approach, where each new tree corrects errors from previous ones using gradient descent optimization. By iteratively refining predictions, XGB enhances model accuracy while minimizing a defined loss function. Its ability to efficiently handle large datasets, missing values, and feature interactions makes it a powerful choice for predictive modeling.

#### Performance evaluation

To evaluate classification performance, metrics suited for imbalanced datasets were chosen, as accuracy alone can be misleading by failing to account for disparities between majority and minority classes. Therefore, sensitivity, specificity, F1-score, and the Area Under the Curve (AUC) were employed. These metrics are commonly used for assessing disease classification models and provide a more comprehensive measure of performance^14,35,36^. Based on the confusion matrix, the evaluation metrics can be evaluated as follows:

$$\text {Precision}=\frac{T_P}{T_P+F_P},\quad \text {Sensitivity}=\frac{T_P}{T_P+F_N},\quad \text {Specificity}=\frac{T_N}{T_N+F_P},\quad \text {F1-score}=2\times \frac{(\text {Precision}\times \text {Sensitivity})}{(\text {Precision}+\text {Sensitivity})}$$

where $T_P$, $T_N$, $F_P$ and $F_N$ denote true positive, true negative, false positive and false negative, respectively. AUC measures the area under the Receiver Operating Characteristic (ROC) curve, which plots 1-specificity on the *x*-axis and sensitivity on the *y*-axis at various classification thresholds. Sensitivity measures the ability to correctly identify severe cases ($T_P$), while Specificity measures the ability to correctly identify non-severe cases ($T_N$). The F1-score, representing the harmonic mean of precision and recall, assesses the model’s accuracy for the positive class, which is particularly useful for imbalanced datasets. Finally, the AUC evaluates the model’s overall discriminative power across all classification thresholds.

### Features’ interpretation

SHAP is a model-agnostic explanation approach with a strong theoretical foundation for evaluating individual predictions^37–39^. Kernel SHAP provides a computationally efficient method to approximate these values, though it operates under the assumption of feature independence. The method aims to measure the impact of each feature on a model’s output. The SHAP value, $\phi _{i}(\nu )$, quantifies this impact for a feature *i*. It is calculated as a weighted average of the feature’s marginal contribution to the model’s expected output, $\nu (S)$, when it is added to all possible subsets of other features, *S*. In this study, the empirical conditional distribution approach was employed to estimate these contributions. Global feature importance is determined by ranking features based on their mean absolute SHAP values. The percent contribution for the top-ranked features, $P(\phi _i(\nu ))$, was then calculated by normalizing the absolute value of each feature’s $\phi _i(\nu )$ against the sum of absolute values for that specific subset.

## Results

This study investigates the impact of various factors on classification model performance. Different resampling techniques, which artificially create training and testing sets, were employed to evaluate model stability. The influence of feature selection, where irrelevant or redundant features are removed, was explored. Finally, the performance of several popular classification algorithms was compared to determine the most effective one for the given task. More detail on specific findings is illustrated in the following subsections.

### Important features

This study addressed data dimensionality in psoriasis classification. LASSO reduced features from 37 to 18, enhancing interpretability and reducing overfitting. MDA accounted for potential heterogeneity, while MDI identified key features. This integrated approach facilitated improved patient stratification and personalized treatment strategies. Table S4 ranks the importance scores for basic demographic, clinical and dietary features based on MDA and MDI technique. Among 10 demographic features, the most important demographic features affecting psoriasis severity appear to be BMI, age, marital status and smoking, respectively. Other features like gender, education, other disease, exercise, cooking oil, and food source seem to have lower importance values for predicting psoriasis severity from 142 patients. Focusing on the eating styles, the MDA and MDI highlighted that FB17-FB24 are significant among the 25-item questionnaire given in Table S2. Applying MDA techniques, the importance scores were varied differently for different training/testing split ratios and it was found that FB17 or alcohol drinking was assigned as the most important one, followed by FB20, FB18, FB24 and so on. For MDI techniques, the importance scores of all items were nearly the same.

### Comparison of classifiers

This comprehensive evaluation aims to optimize model performance by addressing potential pitfalls and identifying the best combination of techniques for the specific dataset and classification problem. As shown in Table S5, the classification performance of 15 RF and 15 XGB classifiers without bootstrapping were evaluated. With the original dataset of size 142, it is seen that all 30 scenarios showed low performance. No matter how feature selection, train/test split ratios and classifiers were combined, all evaluation metrics including sensitivity, specificity and F1-score were considerably low. LASSO shows moderate performance, with varying F1-scores $(38.59-50.00\%)$ for RF and XGB classifiers, depending on the split. Sensitivity and specificity scores fluctuate, indicating inconsistent model performance across splits. MDA provides a higher specificity $(75.00-85.00\%)$ than sensitivity $(30.77-47.37\%)$ in RF. For XGB, MDA also demonstrates better specificity than sensitivity but has lower overall F1-scores. MDI performs better in RF with specificity ranging from 58.33% to 81.25% and consistently higher sensitivity in XGB. Given the potential consequences of overlooking severe psoriasis, a high sensitivity is crucial. The highest value of sensitivity is 56.25% obtained from both RF and XGB classifiers with MDI scoring and 75/25 split ratio. The highest-performing model among the 30 tested demonstrated limited effectiveness in capturing severe psoriasis cases, accurately identifying just over half. This indicates a clear necessity for further refinement and optimization.

### Influence of bootstrapping

**Fig. 3:**
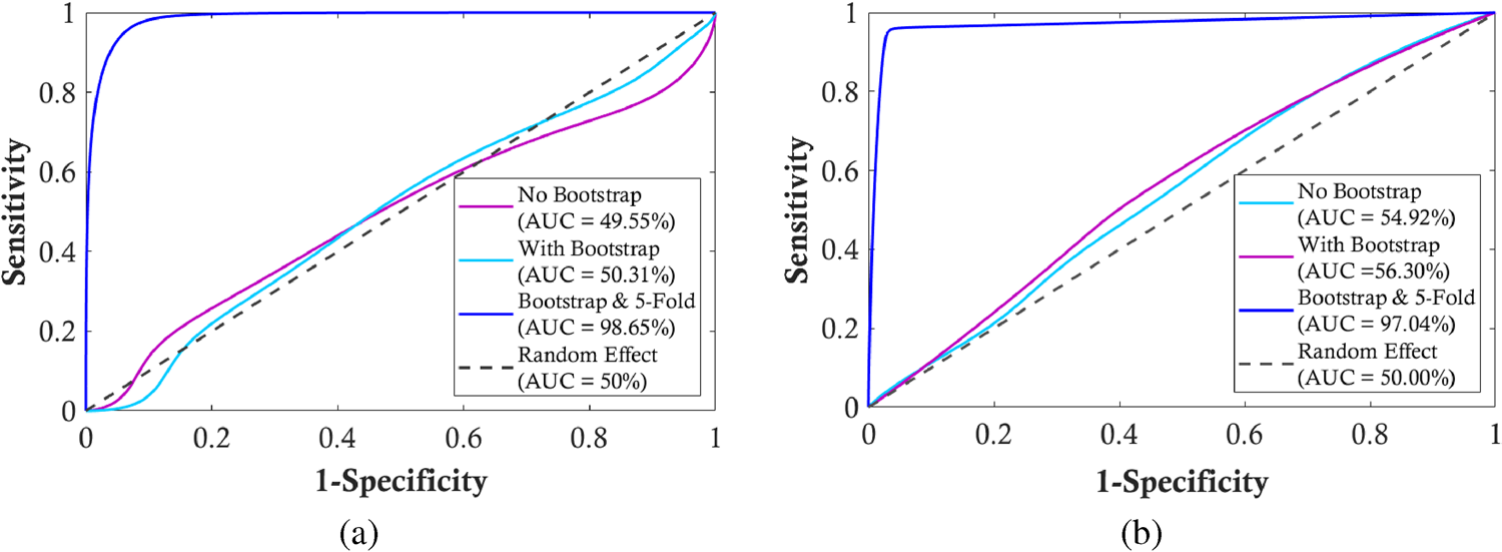
Receiver Operating Characteristic (ROC) curves for the **a** Random Forest (RF) and **b** eXtreme Gradient Boosting (XGB) classifiers in predicting psoriasis severity. Each plot compares the model’s performance under three conditions: without bootstrapping, with bootstrapping only, and with bootstrapping combined with 5-fold cross-validation. The Area Under the Curve (AUC) is reported for each condition, with the random effect line (AUC = 50%) as the reference.

Increasing the sample size by bootstrapping techniques and setting $n_{\text {train}}$ $=375$, the classifiers were re-executed. The testing sets were fixed as the original one as in Table S3 and model performances were re-evaluated. As shown in Table S5, the classifiers with *K*-fold CV present their high performance whereas the classifiers with 80/20, 75/25 and 70/30 split ratios still show poor performance. Additionally, the classification performance of 60 different scenarios were investigated: LASSO, MDA, and MDI methods for RF and XGB classifiers with and without bootstrapping, across different train/test splits and CV folds. Introducing the bootstrap method into the experimentation, LASSO dramatically improves with bootstrapping, achieving high specificity $(96.40 - 99.99\%)$ and F1-scores $(91.78 - 92.18\%)$ for RF. Similarly, XGB also shows improved performance. MDA benefits from bootstrapping, achieving high sensitivity $(90.73 - 91.63\%)$ and specificity $(95.21-95.81\%)$, and good F1-scores $(90.99-92.43\%)$ for both classifiers. MDI performed the most consistent improvement with bootstrapping, achieving near-perfect sensitivity, specificity, and F1-scores in RF and XGB classifiers. Particularly, both RF and XGB classifiers with either 5-fold or 10-fold CV provided the recall of approximately $90.00\%$ over LASSO, MDA and MDI feature selections. To visualize the classification performance, Fig. 3 presents the AUC values for two different classifiers including RF and XGB to predict psoriasis severity. With the addition of both bootstrapping and 5-fold CV, the RF’s performance significantly improves, achieving an AUC of $98.65\%$. For XGB, the most considerable enhancement occurs with the combination of bootstrapping and *K*-fold CV, where the AUC rises to $97.04\%$. Thus, the application of both bootstrapping and 5-fold CV significantly improves their performance, resulting in high AUC values close to 1.

### SHAP for features’ contribution

After the best model was found (RF classifier with bootstrapping and *K*-fold CV), SHAP values were evaluated for all LASSO-selected features ranked by MDA and MDI techniques. Global explanations reveal the most influential features across the population while local explanations demonstrate how individual-level factors affect model predictions. The global and local explanation are illustrated as follows.

#### Global explanation of RF model

**Fig. 4:**
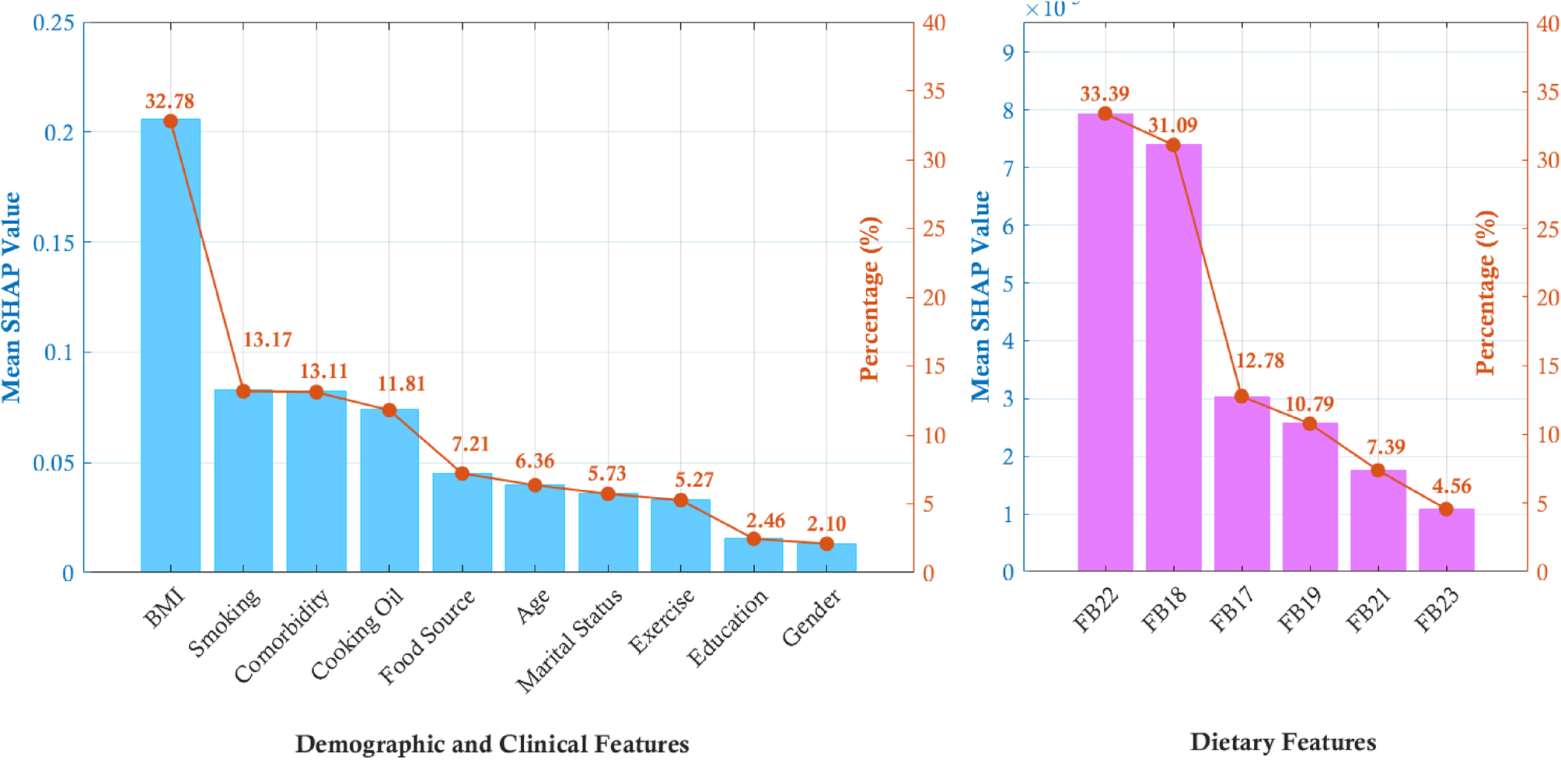
Global feature importance for the prediction of psoriasis severity, derived from the best-performing Random Forest model. The plots display the mean absolute SHAP (SHapley Additive exPlanations) values, which represent the average magnitude of a feature’s impact on the model’s prediction. The top-ranking features are separated into: **a** Demographic and clinical features, and **b** Dietary features. The model was trained using MDI-based feature selection, 5-fold cross-validation, and bootstrapping.

**Fig. 5:**
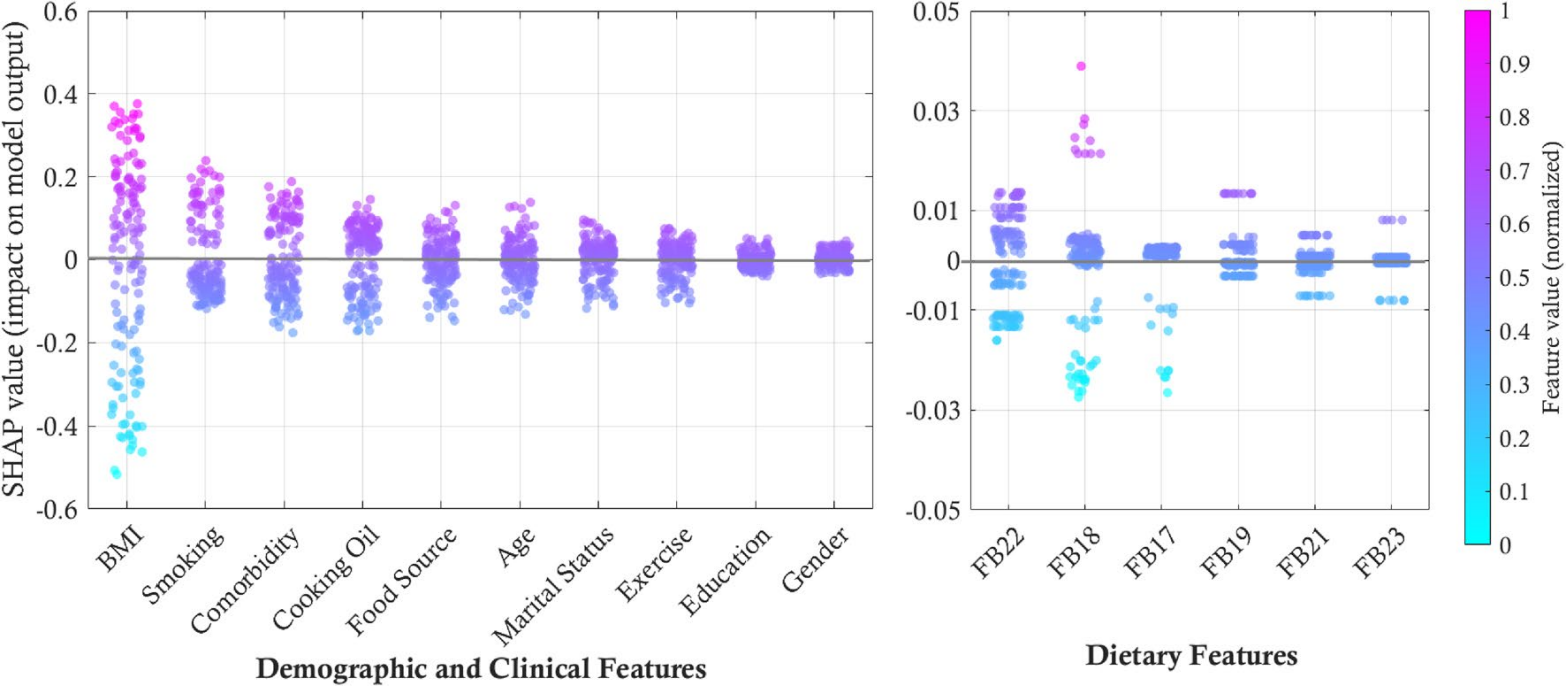
SHAP (SHapley Additive exPlanations) summary plots illustrating the impact of individual features on the model’s prediction of psoriasis severity. The plots are derived from the best-performing Random Forest model. Each point on the plot represents a single patient, where its horizontal position indicates the feature’s impact on the model output (SHAP value), and its color represents the original feature value (high values in magenta, low values in cyan).

As shown in Fig. 4, the contribution of various features to the severity of psoriasis, as measured by average absolute SHAP values is visualized. The percentages represent the extent to which each feature contributes to the model’s predictions regarding psoriasis severity. BMI (32.78%), smoking (13.17%) and comorbidity (13.11%) are the top three contributors to higher psoriasis severity, followed by cooking oil, food source, age, marital status, exercise, education and gender, respectively. Among all features, BMI emerged as the strongest predictor of psoriasis severity, underscoring its critical role in disease progression. Furthermore, various food categories exhibit differing impacts, highlighting the need for targeted dietary modifications in managing psoriasis. *Chili and seasonings* (FB22) contribute the highest proportion, accounting for 33.39% of dietary influence as most Thai menus have high sodium and strong flavored. Culturally, various chili condiments and seasoning are often served with Thai foods after cooking. Comparably, *Processed meats* (FB18) show the second ranking for 31.09%. Foods like sausages, pickled fish, and bouncy meatballs, common in Thai cuisine, are rich in additives and preservatives that exacerbate systemic inflammation, aggravating psoriatic symptoms. *Alcohol consumption* (FB17), at 12.78%, is associated with heightened psoriasis severity due to its pro-inflammatory effects and disruption of the skin barrier. *Red meats* (FB19) follow with 10.79%, as frequent consumption of pork, lamb, and beef is linked to psoriasis severity due to their saturated fats and pro-inflammatory compounds. *Fermented foods* (FB21) account for 7.39%, influencing gut microbiota and systemic inflammation. Lastly, *Dark colored vegetables* (FB23) exhibit an average absolute SHAP value of 4.56%, indicating a low contribution to the model. Without taking the absolute value, the SHAP value is - 0.0128, suggesting a slightly negative effect; however, this direction is not strong enough to confirm a definitive influence on psoriasis severity. Clinically, vegetables are still recommended for patients due to their high nutritional value and the potential to alleviate symptoms of psoriasis. Additionally, the impact of individual features on the model’s prediction of psoriasis severity is shown in Fig. 5 suggesting that demographic and clinical features contribute more substantially to model predictions than dietary features. Clinically, these findings suggest that weight management should be a priority in psoriasis care, with BMI being the strongest predictor of disease severity. To reduce flare-ups and support long-term disease control, dietary counseling should target key contributors to inflammation including high-sodium seasonings, processed meats, alcohol, red meats and fermented foods.

#### Local explanation of four random patients

Taking into account the local SHAP analyses, four representative patients were exemplified in Figs. S2 and S3, demonstrating how demographic, clinical, and dietary variables contribute to modeled psoriasis severity. In Fig. S2, demographic and clinical factors (age, BMI, smoking, exercise, comorbidity) show individualized positive or negative effects on predictions. Fig. S3 highlights analogous contributions from dietary features. Positive SHAP values drive predictions towards higher severity, whereas negative values reduce them. Cases 10 and 37 show dominant positive effects $(Y=1)$, while Cases 22 and 88 show negative contributions $(Y=0)$. Individually, Case 10 exhibits potential demographic and clinical feature while demonstrating healthy dietary behaviors that are unlikely to exacerbate psoriasis severity. Despite good dietary control, the very high BMI (39.5421) is a major risk factor for psoriasis flare-ups and reduced treatment efficacy. The patient should consider a weight management program. Case 37 presents with significant demographic and clinical risk factors, along with unhealthy dietary behaviors that may contribute to increased psoriasis severity so that diet modification is urgent. Within the lower psoriasis severity group, Case 22 exhibits poor dietary habits whereas Case 88 demonstrates favorable demographic, clinical, and dietary profiles. Despite low psoriasis severity, the presence of comorbidities and poor dietary habits necessitates early intervention.

## Discussion

This section discusses the challenges of high dimensionality and small sample size in classifying psoriasis severity, along with the advantages of the proposed embedded techniques, including LASSO, MDA, MDI, bootstrapping and *K*-fold CV in enhancing model performance, interpretability, and generalization.

### Classification model

Analysing the relationship between dietary pattern and psoriasis severity, this study have met two major challenges in the dataset obtained including high dimensionality as well as the interpretability towards real-world implementation. Each issue is discussed as follows:

#### High dimensionality reduction

From an initial set of 37 features, dimensionality reduction was performed by LASSO and a subset of 18 predictors were then obtained. Classifying binary psoriasis severity (PASI $< 10$ vs. PASI $\ge 10$), the importance of these features was further assessed using MDA and MDI. The original dataset ($n=142$) presented a low event-per-predictor (EPP) ratio of 3.8378 (142/37)^23,24^. To address this limitation, a hybrid approach combining bootstrapping and *K*-fold CV was employed. This strategy increased the effective training size to 375 and improved the EPP to values ranging from 11.1622 to 12.1622 under various resampling conditions. Although these techniques were essential for mitigating the effects of the small sample size, findings indicated that the resulting improvements in classification performance were still constrained by the inherent data limitations.

#### Model robustness

A combination of bootstrapping and *K*-fold CV was employed to enhance model reliability. The resulting training size ($90 \times 4 = 360$ for $K = 5$, and $40 \times 9 = 360$ for $K = 10$) satisfies the rule of thumb for prediction modeling, with events per predictor ($\text {EPP} = 360/18 = 20 \ge 10$). This approach led to a substantial improvement in predictive performance compared to models trained without bootstrapping (Table S5), consistent with findings from prior simulation studies^23,40^. To prevent overfitting and ensure model generalizability, additional regularization and validation procedures were applied. Nested *K*-fold CV was implemented, wherein hyperparameters were optimized in inner folds and evaluated in outer folds to avoid information leakage. *L*1 regularization (LASSO) was used for feature selection, retaining only clinically plausible predictors. Biological plausibility was evident in the top features: baseline PASI, BMI, and FB22 (high-sodium diet), which align with findings from recent multicenter studies^41,42^, suggesting a true signal rather than overfitting or noise. Despite a modest cohort size, the final model adheres to accepted sample size recommendations for predictive modeling and is further supported by robust validation techniques. Nevertheless, future models should incorporate additional predictors to enhance performance and improve external validity.

#### Feature importance

Classifying psoriasis severity, feature importance plays a pivotal role in identifying influential variables. Implementing MDA and MDI, both of them have strengths and limitations when applied to complex medical datasets like psoriasis severity. The dietary patterns significantly influence psoriasis severity, adding another layer of complexity. The proposed questionnaires and ML models struggled to capture the intricate relationship between specific diets and PASI scores. Variability in individual dietary habits, combined with other factors like genetics and environment, complicates severity classification. Therefore, more comprehensive clinical research is essential to develop improved models that account for dietary influences, enhancing prediction accuracy in healthcare settings.

### Dietary patterns and psoriasis severity

Diet plays a critical role in managing psoriasis, a chronic inflammatory condition influenced by immune and metabolic factors. Certain foods can exacerbate inflammation, while others possess anti-inflammatory properties that may alleviate symptoms. This section explores the potential connections between dietary styles, food groups, and specific nutrients with the development and severity of psoriasis. Different dietary patterns and the intake of certain nutrients may impact the inflammatory processes associated with psoriasis.

#### Influence of dietary patterns

Understanding the impact of specific dietary components provides valuable insights into how nutrition affects psoriasis severity and progression. This discussion explores evidence-based findings on key food groups, emphasizing their role in systemic inflammation, immune modulation, and practical dietary recommendations to support better psoriasis management and overall health outcomes. *Capsaicin* in chili (FB22), the active compound in chili peppers, exhibits a dichotomous effect on psoriasis; while it possesses anti-inflammatory properties, it can also induce flare-ups by elevating body temperature and causing sweat-induced irritation^43^. Thai cuisine is typically prepared with abundant seasonings and served with a variety of chili-based condiments and various sauces such as Nam Prik, Nam Chim, Sriracha and etc. These condiments are often high in sodium, which may exacerbate psoriasis symptoms in sensitive individuals^12,44–46^. *Processed meats* (FB18), such as sausages and pickled fish, contain high levels of salt, refined carbohydrates, and saturated fats, contributing to systemic inflammation and immune dysregulation^44–46^. High-sodium diets activate pro-inflammatory cytokines (IL-17, TNF-$\alpha$), exacerbating psoriatic lesions, while nitrates and preservatives induce oxidative stress, impairing skin barrier function. *Alcohol consumption* (FB17) aggravates psoriasis by stimulating keratinocyte proliferation, promoting pro-inflammatory cytokines (TNF-$\alpha$, IL-6), and impairing liver function, reducing detoxification of inflammatory mediators^6,47,48^. Women with higher alcohol intake report more severe symptoms. *Red meat* (FB19), including beef, lamb, and pork, is rich in arachidonic acid, which metabolizes into inflammatory mediators, worsening psoriasis^6,9^. Saturated fats activate the NLRP3 inflammasome and IL-23/IL-17 pathway, while heme iron contributes to oxidative stress. In Thai patients, high red meat and belly meat intake correlates with increased psoriasis severity. *Fermented or pickled foods* (FB21), high in sodium, influence gut microbiota and immune function. Sodium-rich diets worsen psoriasis by activating Th17 cells and disrupting gut microbial diversity, increasing intestinal permeability and systemic inflammation^44–46^. *Dark colored vegetables* (FB23), such as spinach and kale, are rich in vitamin E and beta-carotene, which neutralize free radicals and downregulate IL-17 and TNF-$\alpha$, reducing oxidative stress and inflammation^7,8^.

**Fig. 6:**
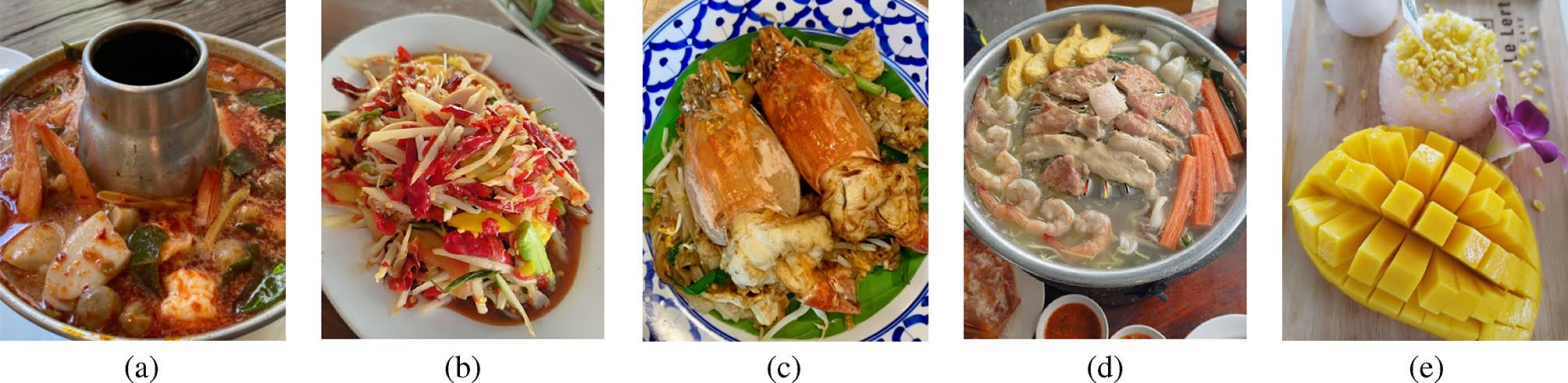
Examples of popular Thai dishes containing potential dietary triggers that may exacerbate psoriasis symptoms. These dishes often feature common inflammatory triggers such as chili, shellfish, and fatty meats. Shown are: **a** Tom Yum Kung (Spicy shrimp soup); **b** Som Tum (Spicy papaya salad); **c** Pad Thai (Stir-fried rice noodle); and **d** Moo Kratha (Thai Barbecue); **e** Khao Niao Mamuang (Mango sticky rice).

#### Popular Thai cuisines associated with psoriasis triggers

Thai cuisine is celebrated for its unique flavors, fresh ingredients, and nutritional value. It features herbs and spices like lemongrass, turmeric, chili, and ginger, known for their antioxidant and anti-inflammatory properties^49,50^. Thai dishes provide a balanced mix of “*good*” carbohydrates, lean proteins, and fiber-rich vegetables, supporting digestion and immune health. With its focus on fresh, nutrient-dense components, Thai meals often avoid excessive processed condiments that can increase inflammation. This makes Thai cuisine a suitable choice for anti-inflammatory diets, offering flavorful, healthy options that promote overall well-being, and potentially alleviating symptoms in conditions linked to inflammation, such as psoriasis. The four popular Thai recipes are exemplified in Fig. 6. Thai cuisine, particularly *Tom Yum Kung*, is rich in anti-inflammatory herbs such as lemongrass, galangal, and kaffir lime leaves, which contain bioactive compounds with antioxidant and immune-modulating properties that may help reduce inflammation and alleviate symptoms^51–54^. These ingredients have been associated with lowering oxidative stress and supporting immune function, making them beneficial for individuals with inflammatory conditions. However, traditional dishes such as *Somtum*, *Pad Thai*, *Moo Kratha* and *Khao Niao Mamuang* often contain high levels of fat, sodium, and refined carbohydrates, which may contribute to metabolic imbalances and increased inflammation. Given Thailand’s global reputation for diverse and flavorful street food, individuals with psoriasis (both locals and tourists) should exercise caution when selecting Thai dishes, as many traditional recipes may contain dietary triggers. While Thai cuisine is widely appreciated for its taste, modifications to ingredients may be necessary to reduce potential flare-ups. For instance, Som Tum should be prepared with minimal monosodium glutamate (MSG) and fermented fish sauce; Pad Thai should be cooked with less oil and reduced seasoning; and Moo Kratha should emphasize a higher proportion of vegetables while limiting high-fat meats such as streaky pork or bacon. When ingredient customization is not feasible, it is advisable for psoriasis patients to limit the consumption of these dishes, especially Khao Niao Mamuang. Dietary choices should be informed by awareness of specific triggering ingredients, as identified in this study, to better manage psoriasis symptoms through nutrition.

#### Specific nutrients and food recommendation

Research on specific nutrients highlights the role of macronutrients and micronutrients in psoriasis management. Reducing caloric intake, particularly carbohydrates, proteins, and fats, alleviates symptoms^6–9,12,41^. Simple sugars, meat-derived proteins, saturated fats, and alcohol are known to exacerbate psoriasis by promoting systemic inflammation through mechanisms such as inflammasome activation, reactive oxygen species generation, and regulatory T cell suppression^6,12,47,48^. Consistent with these mechanisms, our study found associations between higher psoriasis severity and the consumption of high-sodium foods, processed and red meats (e.g., pork, lamb, buffalo, beef), alcoholic beverages, and fermented foods. In contrast, unsaturated fats demonstrate anti-inflammatory effects and are beneficial for psoriasis management. Micronutrients also play a critical role in modulating disease activity. Antioxidant vitamins A, B12, C, and E contribute to skin health and immune regulation, while vitamin D provides immunomodulatory and antiproliferative benefits in deficient individuals^6–8,12^. Selenium has shown efficacy in improving psoriatic lesions by supporting regulatory T cell activation and suppressing inflammatory pathways. Omega-3 polyunsaturated fatty acids from fatty fish such as salmon and mackerel have been associated with reduced inflammation and improved psoriasis outcomes. However, the presence of environmental contaminants such as mercury and dioxins in some seafood may counteract these benefits^7–9^. Similarly, fruits offer a rich source of antioxidants, vitamins, and polyphenols that mitigate oxidative stress and systemic inflammation^9^. Citrus fruits and berries, in particular, are high in vitamin C, which supports collagen synthesis and skin repair, contributing to symptom reduction. Dietary strategies should be personalized according to individual health profiles^55^. In the Thai context, recommendations must consider traditional dishes often rich in sodium, sugar, and saturated fats. Ingredients such as fermented fish sauce, shrimp paste, and chili-based condiments can trigger symptoms in sensitive individuals. Psoriasis patients are encouraged to choose lower-risk options such as *Tom Yum Kung* or steamed fish, and to limit dishes like *Som Tum*, *Pad Thai*, and *Moo Kratha* unless modified. Healthier substitutions (e.g., brown rice instead of jasmine rice, tofu or tempeh in place of red meat, and greater use of fresh herbs and vegetables) can support anti-inflammatory dietary goals. Increased fruit and vegetable intake remains universally beneficial^7,8^. However, some tropical Thai fruits (e.g., rambutan, longan, durian, mango) though rich in micronutrients, have high glycemic indices and fructose content that may promote inflammation and impair glycemic control, contributing to psoriasis exacerbation. Traditional Thai desserts like *Khao Niao Mamuang* (Mango sticky rice), which combine high-sugar fruits with refined carbohydrates, should be consumed in moderation by individuals with psoriasis. Gluten-free diets are only effective in patients with confirmed gluten sensitivity or celiac disease, while vitamin D supplementation is advisable in cases of deficiency^12^. For patients with elevated BMI, a low-calorie, nutrient-dense diet is recommended, as weight loss has been shown to improve psoriasis severity and treatment response^41^. Finally, reducing or abstaining from alcohol is critical, as alcohol not only heightens systemic inflammation but also interferes with drug metabolism and may reduce treatment efficacy^6,12,47,48^.

### Implementation

The proposed framework addresses key challenges in classifying psoriasis severity while offering valuable insights for broader healthcare applications aimed at improving patients’ QoL. Central to this approach are health literacy and smart healthcare technologies, which empower patients to make informed dietary decisions and adhere to anti-inflammatory recommendations. Integrating culturally tailored education, digital health tools, and ML-driven personalization, the framework supports real-time symptom monitoring, identification of dietary triggers, and individualized care.

#### Health literacy in psoriasis dietary management

Clinicians and patients can assess and enhance health literacy by focusing on understanding the nutritional components and inflammatory potential of specific foods. Education should emphasize how macronutrients, micronutrients, and traditional Thai ingredients influence psoriasis. Clear guidance on modifying high-risk dishes, identifying beneficial substitutions, and tailoring diets to individual needs ( e.g., elevated BMI, gluten sensitivity, or vitamin deficiencies) can empower patients to make informed dietary choices. Health literacy encompasses the ability to interpret food labels, understand dietary recommendations, and apply them within one’s cultural eating patterns. Collaborative clinician-patient dialogue is essential to bridge knowledge gaps and promote sustainable, anti-inflammatory dietary habits for psoriasis management. The findings from this study emphasize the critical role of patient education in linking dietary behaviors to psoriasis severity. Evidence shows that increased consumption of red meat, processed foods, and alcohol correlates with higher disease severity (PASI$\ge$10), whereas anti-inflammatory diets rich in fruits, vegetables, and omega-3 fatty acids are associated with symptom improvement^8,55,56^. To enhance adherence, educational efforts should leverage culturally adapted programs, digital health tools, and community support. Mobile applications such as “*Psoriasis Diary*” offer real-time tracking and personalized feedback, further enabling patients to manage their condition effectively.

#### Health management and smart healthcare technology

ML models enable predictive risk stratification and personalized care. Multi-disciplinary approaches involving dermatologists, dietitians, and primary care providers improve outcomes^56,57^. Integrating Thai dietary habits into care protocols ensures culturally sensitive interventions. Digital tools further support dietary tracking and education. Smart healthcare systems leverage ML algorithms for real-time data analysis and personalized interventions. Digital health platforms, AI-powered nutrition guidance, and telemedicine enhance patient engagement and improve psoriasis management^13,14,16,17,22^. Focusing on the personalized psoriasis management, demographic, clinical and dietary features should be monitored and determined whether one is potential to psoriasis severity. The impact of individual features and kernel SHAP force should be introduced to such digital health platforms. Integrating wearable devices for symptom and lifestyle monitoring, mobile apps for food logging, and AI chatbots for real-time guidance enhances patient engagement. These technologies empower patients to take active roles in managing their psoriasis, improve adherence to anti-inflammatory dietary recommendations, and enable early identification of symptom triggers.

### Limitations

Some limitations should be considered when interpreting these results. The relatively small sample size $(n=142)$, combined with notable heterogeneity in age (19-74 years) and lifestyle, may constrain statistical power and increase the likelihood of overfitting despite cross-validation procedures. Furthermore, the cross-sectional design limits causal inference, permitting only the identification of associations between dietary patterns and psoriasis severity. It is also important to consider population-specific factors that may influence these findings. The Thai population is characterized by genetic backgrounds that differ from Western cohorts, including variations in Human Leukocyte Antigen (HLA) alleles and immune response genes previously linked to psoriasis susceptibility^3,58^. Moreover, Thailand’s tropical climate, with consistently high temperatures and humidity, alongside regionally distinct dietary customs, seasonal food availability, and cultural practices, may interact with disease expression and nutritional behaviors. These environmental and genetic contexts should be considered when interpreting the applicability of our findings to other populations. As the cohort comprised Thai patients, generalizability to other populations may be restricted. Nevertheless, these findings offer a meaningful basis for future validation in larger, multi-center prospective studies. The more advanced approaches dealing with small-sample size such as Bayesian framework should be further experimented.

## Conclusions

The integration of ML approaches into psoriasis severity assessment addresses challenges of small sample sizes compared to a number of features. To satisfy the rule of thumb (EPP$\approx$10), resampling techniques including bootstrapping and $K$-fold CV, enhanced dataset robustness, increasing training size to $n_{\text {train}} = 375$. Feature selection using LASSO, MDA and MDI refined the model by identifying and reducing key predictors from 37 to 16 features. LASSO-selected features included demographic, clinical and dietary features offering insights into potential factors of psoriasis severity. RF and XGB classifiers demonstrated significant improvements with the hybrid resampling strategy. RF with MDI scoring, combined with 5-fold CV and bootstrapping, achieved sensitivity and specificity exceeding 90%, validated by ROC curves with AUC values nearing 1. The findings have practical implications for personalized healthcare, with identified predictors guiding dietary interventions and public health strategies. Clinically, prioritizing weight management is essential, as BMI emerged as the strongest predictor of psoriasis severity. Dietary triggers identified in this study should be incorporated into comprehensive care plans. Reducing high sodium foods, processed meat, alcohol drinks and red meat while promoting antioxidant-rich foods (e.g., fish, fruits, vegetables) may mitigate severity. Thai cuisine, particularly *Tom Yum Kung*, is abundant in anti-inflammatory herbs, which may contribute to symptom relief. However, high-fat or high-sodium dishes, such as *Som Tum*, *Pad Thai*, *Moo Kratha*, and *Khao Niao Mamuang* should be consumed in moderation. Beyond psoriasis, this methodological framework can be applied to similar small-sample or high-dimensional datasets in medical research. In conclusion, ML coupled with the effective resampling technique, feature selection methods, and robust classifiers, enhances psoriasis management while providing actionable insights for clinical practice and dietary recommendations. Future applications include integrative health literacy, health management, and smart healthcare technologies.

## Supplementary Information

Supplementary Information.

## Data Availability

The dataset utilized in this study is available on GitHub at https://github.com/strawat/Thai_Psoriasis_data, containing all essential data files and documentation to ensure reproducibility. Researchers incorporating this dataset in their work are encouraged to cite this study to acknowledge its contribution.
