# Supplementary material for: Dietary patterns and psoriasis severity in Thai patients: a machine learning approach for small sample data
Source: Sci Rep. 2025 Sep 26;15:33088. doi: 10.1038/s41598-025-17657-z (PMC12475393; doi:10.1038/s41598-025-17657-z)
Supplement: Supplementary file 1 — Supplementary Information. [file 41598_2025_17657_MOESM1_ESM.pdf]

Supplementary Materials

Questionnaire on Dietary Pattern in Psoriasis Patient Patient ID.___________

This questionnaire assesses demographic, clinical, and dietary factors to identify potential correlates of psoriasis severity. Please answer each question carefully and accurately.

Part I: Patient's Demographics

Direction: Please fill key demographic and clinical data for establishing a baseline profile for clinical observation.

Q1. Age: How old are you ? [__________] years

Q2. BMI:
Weight: [__________] kg. Height: [__________] cm.

Please state your BMI: [__________]

Q3. Severity of Psoriasis (PASI):
PASI score evaluated by clinician: [__________]
☐ 0: PASI < 10 ☐ 1: PASI ≥ 10

Q4. Gender:
☐ 1: Male ☐ 2: Female

Q5. Marital status:
☐ 1: Single ☐ 2: Married ☐ 3: Others

Q6. Education: What is your highest educational level ?
☐ 1: Below Undergraduate
☐ 2: Undergraduate or Higher

Q7. Comorbidity:
☐ 0: No other disease ☐ 1: With some diseases

Q8. Smoking:
☐ 0: No ☐ 1: Yes ☐ 2: Previous smokers

Q9. Exercise: Do you exercise $\geq$2 times/week ?
☐ 0: No ☐ 1: Yes

Q10. Taste preferences:
☐ 1: Sweet ☐ 2: Spicy ☐ 3: Salty
☐ 4: Sour ☐ 5: Plain ☐ 6: Others

Q11. Cooking Oil:
☐ 1: Lard ☐ 2: Palm ☐ 3: Soybean
☐ 4: Coconut ☐ 5: Others

Q12. Food source:
☐ 1: Self Cooking ☐ 2: Buy from market
☐ 3: Eat at a restaurant ☐ 4: Ready-to-eat food
☐ 5: Others

Q13. Food selection criteria:
☐ 1: Cheap ☐ 2: Good quality ☐ 3: Popular
☐ 4: Delicious ☐ 5: Others

Part II: Typical Food and Drink Consumption Behavior

Direction: Please consider how often you consume the specified food for each item. Use the frequency scale to best represent your usual habits (**0**: Never, **1**: 1-2 times/two week, **2**: 1-2 times/week, **3**: $\geq$3 times/week).

Item	Food Consumption	0	1	2	3
**FB01**	How often do you have more than one serving of rice or noodles per meal ?				
**FB02**	How often do you have more than three meals daily ?				
**FB03**	How often do you have fatty meat (Crispy pork belly, Braised pork leg, Mu Hong, Chicken Rice, etc.) ?				
**FB04**	How often do you have fast foods (Fried chicken, French fries, Pizza, etc.) ?				
**FB05**	How often do you have convenient foods (Instant noodle, Canned fish, etc.) ?				
**FB06**	How often do you have grilled, smoked, or fried food using high heat ?				
**FB07**	How often do you have raw or uncooked meat (Goi Nuea, Soi Ju, Gung Chae Nam Pla, etc.) ?				
**FB08**	How often do you have Thai desserts (Mango sticky rice, Thong Yip, Ruam Mit, Shaved ice, etc.) ?				
**FB09**	How often do you have sweet fruits (Durian, Jackfruit, Longan, Sapodilla, Mango, etc.) ?				
**FB10**	How often do you have bakeries (Cakes, Donuts, Hamburgers, Sandwich, etc.) ?				
**FB11**	How often do you have fried snacks (Roti, Patongko dough, Spring rolls, Fried bananas, etc.) ?				
**FB12**	How often do you have crispy snacks (Potato chips, Khaep Mu, Rice Crackers, Fried insects, etc.) ?				
**FB13**	How often do you have fizzy beverages (Cola, Soda water, Fruit-flavored soda, etc.) ?				
**FB14**	How often do you have tea or coffee (Cha Thai, Oliang, Bubble milk tea, etc.) ?				
**FB15**	How often do you have milk or milk products (Nom Yen, Cream, Yogurt, Cheese, etc.) ?				
**FB16**	How often do you have soy milk or soybean menus (Tempeh, Tofu, Miso, Tamari) ?				
**FB17**	How often do you have alcoholic beverages (Beer, Wine, Baijiu, Sato, Soju, etc.) ?				
**FB18**	How often do you have processed meat (Sausage, Pickled fish, Meatballs, Sai Krok Isaan, etc.) ?				
**FB19**	How often do you have red meat (Pad Krapow, Steak, Pork, Lamb, Beef, Buff, etc.) ?				
**FB20**	How often do you have fish and seafood (Sea bass, Tilapia, Tuna, Crab, Squid, Shrimp, etc.) ?				
**FB21**	How often do you have fermented foods (Plara, Kapi, Pickled fruits, Pickled vegetables, etc.) ?				
**FB22**	How often do you have chili and seasoning (Nam Prik, Nam Chim, Chili sauces, Thai curry paste, etc.) ?				
**FB23**	How often do you have dark colored vegetables (Morning glory, Spinach, Eggplant, Tomato, etc.) ?				
**FB24**	How often do you have healthy fruits (Guava, Papaya, Pomelo, Watermelon, Passion fruit, etc.) ?				
**FB25**	How often do you have eggs (Fried egg, Omelet, Steamed egg custard, Son-in-law egg, Egg tofu, etc.) ?				

No.	Feature	Description	Type	Range/ Data Label	Mean ± SD./ Count (%)
1	AGE	Age	Numeric	$19-74$	42.25 ± 11.35
2	BMI	Body Mass Index	Numeric	$16.64-45.91$	25.80 ± 5.53
3	SEV	Severity	Binary	0: PASI $<$10	79 (55.63)
				1: PASI $\geq$ 10	63 (44.37)
4	GEN	Gender	Binary	1: Male	77 (54.23)
				2: Female	65 (45.77)
5	MAS	Marital Status	Nominal	1: Single	56 (39.44)
				2: Married	60 (42.25)
				3: Others	26 (18.31)
6	EDU	Education	Binary	1: Below Undergraduate	74 (52.11)
				2: Undergraduate and Higher	68 (47.89)
7	COM	Comorbidity	Binary	0: No Other Disease	86 (60.56)
				1: With Some Diseases	56 (39.44)
8	SMO	Smoking	Nominal	0: No	94 (66.20)
				1: Yes	20 (14.08)
				2: Previous Smokers	28 (19.72)
9	EXE	Exercise	Binary	0: No	82 (57.75)
				1: Yes	60 (42.25)
10	TAS	Favorite Taste	Nominal	1: Sweet	30 (21.13)
				2: Spicy	33 (23.24)
				3: Salty	22 (15.49)
				4: Sour	21 (14.79)
				5: Plain	20 (14.08)
				6: Others	16 (11.26)
11	OIL	Cooking Oil	Nominal	1: Lard	24 (16.90)
				2: Palm	59 (41.55)
				3: Soybean	41 (28.87)
				4: Coconut	10 (7.04)
				5: Others	8 (5.63)
12	FOS	Food Source	Nominal	1: Self Cooking	64 (45.07)
				2: Buy from Market	38 (26.76)
				3: Eat at a Restaurant	27 (19.01)
				4: Ready-to-eat Food	13 (9.15)
13	PRI	Food Selection	Nominal	1: Cheap	2 (1.41)
				2: Good Quality	53 (37.32)
				3: Popular	2 (1.41)
				4: Delicious	53 (37.32)
				5: Others	32 (22.54)

Table S1. A summary of baseline characteristics for the 142 psoriasis patients in the study. The table details demographic, clinical, and lifestyle features. Data are presented as mean ± standard deviation for continuous variables, and as frequency counts and percentages for all categorical variables.

Item	Consumption	0 (%)	1 (%)	2 (%)	3 (%)
FB01	More than one serving of rice or noodles per meal	29 (20.42)	54 (38.03)	40 (28.17)	19 (13.38)
FB02	More than three meals daily	54 (38.03)	45 (31.69)	26 (18.31)	17 (11.97)
FB03	Fatty meats	19 (13.38)	52 (36.62)	56 (39.44)	15 (10.56)
FB04	Fast foods	39 (27.46)	81 (57.04)	15 (10.56)	7 (4.93)
FB05	Convenient foods	40 (28.17)	71 (50.00)	23 (16.20)	8 (5.63)
FB06	Grilled, smoked, or fried food using high heat	15 (10.56)	62 (43.66)	43 (30.28)	22 (15.49)
FB07	Raw or uncooked meat	93 (65.49)	42 (29.58)	6 (4.23)	1 (0.70)
FB08	Thai desserts	30 (21.13)	68 (47.89)	26 (18.31)	18 (12.68)
FB09	Sweet fruits	23 (16.20)	83 (58.45)	28 (19.72)	8 (5.63)
FB10	Bakeries	43 (30.28)	71 (50.00)	17 (11.97)	11 (7.75)
FB11	Fried snacks	40 (28.17)	71 (50.00)	21 (14.79)	10 (7.04)
FB12	Crispy snacks	37 (26.06)	56 (39.44)	35 (24.65)	14 (9.86)
FB13	Fizzy beverages	50 (35.21)	49 (34.51)	26 (18.31)	17 (11.97)
FB14	Tea or coffee	27 (19.01)	33 (23.24)	23 (16.20)	59 (41.55)
FB15	Milk or milk products	57 (40.14)	51 (35.92)	23 (16.20)	11 (7.75)
FB16	Soymilk or soybean meals	46 (32.39)	55 (38.73)	21 (14.79)	20 (14.08)
FB17	Alcoholic beverages	94 (66.20)	35 (24.65)	9 (6.34)	4 (2.82)
FB18	Processed meat	29 (20.42)	74 (52.11)	29 (20.42)	10 (7.04)
FB19	Red meat	12 (8.45)	29 (20.42)	38 (26.76)	63 (44.37)
FB20	Fish and seafood	5 (3.52)	27 (19.01)	58 (40.85)	52 (36.62)
FB21	Fermented or pickled foods	66 (46.48)	47 (33.10)	24 (16.90)	5 (3.52)
FB22	Chili and seasoning	14 (9.86)	42 (29.58)	51 (35.92)	35 (24.65)
FB23	Dark colored vegetables	1 (0.70)	23 (16.20)	45 (31.69)	73 (51.41)
FB24	Healthy fruits	3 (2.11)	23 (16.20)	44 (30.99)	72 (50.70)
FB25	Eggs	23 (16.20)	54 (38.03)	45 (31.69)	20 (14.08)

Table S2. A summary of responses to the 25-item food consumption behavior (FB) questionnaire from 142 psoriasis patients. The data shows the count and percentage of patients who reported their consumption frequency for each food item. The response categories were defined as follows: 0 (Never), 1 (1-2 times per two weeks), 2 (1-2 times per week), and 3 (three or more times per week).

Validation Technique	Train/Test Splitting							*K*-Fold Cross Validation			
		70/30		75/25		80/20			Each Fold	Class of Y	
Scheme	Class of Y	0	1	0	1	0	1	Scheme	Total	0	1
Without bootstrapping	Train	55	44	59	47	63	50	5-fold CV	28 – 29	15 – 16	12 – 13
	Test	24	19	35	28	16	13	10-fold CV	14 – 15	7 – 8	6 – 7
With bootstrapping	Train	209	166	209	166	209	166	5-fold HR	89 – 90	49 – 50	39 – 40
	Test	24	19	35	28	16	13	10-fold HR	41 – 42	22 – 23	18 – 19

Table S3. A detailed breakdown of the data partitioning used for model development. The distribution of patient samples is presented for two datasets: the original imbalanced dataset ($n_{\text{train}} = 99 - 113$) and the balanced dataset generated via bootstrapping ($n_{\text{train}} = 375$). The number of instances is specified for the two outcome classes (Class 0: PASI$<$10; Class 1: PASI$\geq$10) across two validation techniques: 1) train/test splitting (70/30, 75/25, and 80/20 ratios) and 2) K-fold cross-validation ($K = 5, 10$).

Algorithm S1 MDA (Mean Decrease Accuracy) Scoring

Input: Dataset D containing features X and response variable Y.
Train the **RF** classifier using D and collect out-of-bag (OOB) samples S_B.
Calculate the Baseline Accuracy:
  Predict labels $\hat{Y}$ for S_B using the trained RF.
  Compute baseline accuracy $A_0 = Accuracy(Y, \hat{Y})$.
for each feature X_i **do**
    Permute X_i values in S_B to create $S'_B(X_i)$.
    Predict new labels $\hat{Y}'$ using $S'_B(X_i)$.
    Compute permuted accuracy $A'(X_i) = Accuracy(Y, \hat{Y}')$.
    Compute decrease in accuracy: $\Delta A(X_i) = A_0 - A'(X_i)$.
end for
Compute MDA:
  Average over k permutations: $MDA(X_i) = \frac{1}{k}\sum_{j=1}^{k} \Delta A_j(X_i)$
Output: $MDA(X) = \{MDA(X_1), \ldots, MDA(X_l)\}$, where $l < m$.

Algorithm S2 MDI (Mean Decrease Impurity) Scoring

Input: Dataset D containing features X and response variable Y.
Train the **RF** classifier using D.
Calculate Node Impurity:
  Compute impurity $G(N)$ at each node using the Gini index: $G(N) = 1 - \sum_{i=1}^{c} p_i^2$
for each feature X_i and node N **do**
    Measure impurity decrease:

$$\Delta G(X_i, N) = G(N) - \left(\frac{n_L}{n} G(N_L) + \frac{n_R}{n} G(N_R)\right)$$

    Accumulate impurity decrease across all nodes and trees.
end for
Compute MDI:
  Compute: $MDI(X_i) = \frac{1}{T}\sum_{t=1}^{T}\sum_{N\in t} \Delta G(X_i, N)$
Output: $MDI(F) = \{MDI(X_1), \ldots, MDI(X_l)\}$, where $l < m$.

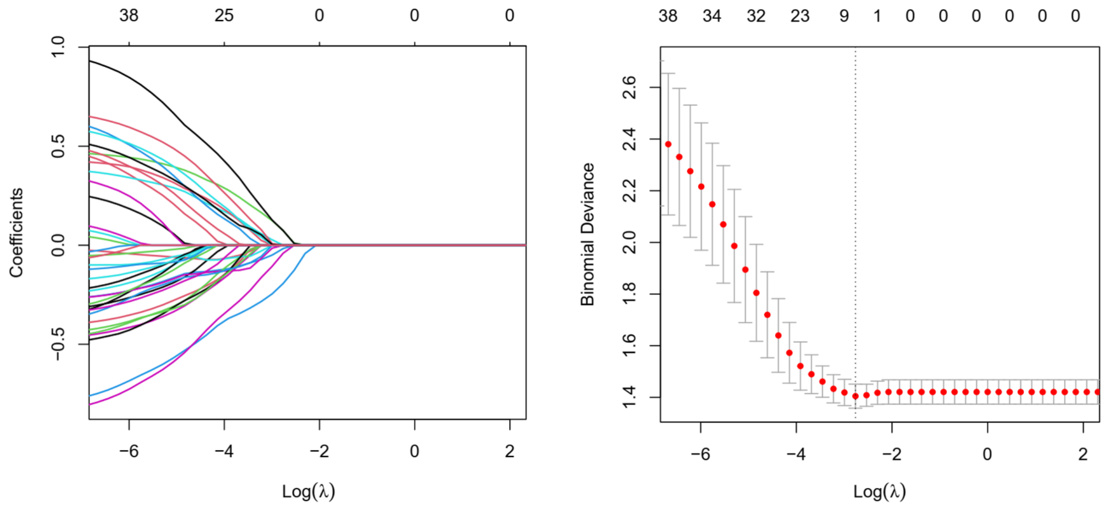

Fig. S1. Regularization path and cross-validation performance for LASSO logistic regression. (Left) Coefficient trajectories for all predictors as a function of the log-scaled regularization parameter (λ), illustrating progressive coefficient shrinkage and variable selection. (Right) Mean binomial deviance with ± 1 SE bars from 5-fold cross-validation plotted against $\log(\lambda)$. The vertical dotted line denotes the λ value achieving the minimum cross-validated deviance, which was used to define the optimal sparse model.

**Feature**	**80/20**		**75/25**		**70/30**	
	**MDA**	**MDI**	**MDA**	**MDI**	**MDA**	**MDI**
Gender	2.762	1.387	3.801	1.446	3.848	1.312
Age	5.941	6.990	6.230	6.375	6.654	6.193
BMI	**12.506**	**10.793**	**12.389**	**10.740**	10.174	**9.603**
Marital Status	9.167	4.167	9.845	4.193	9.543	3.907
Education	3.064	3.179	4.146	3.202	3.971	3.072
Comorbidity	1.439	1.494	3.220	1.922	2.425	1.495
Smoking	8.527	3.077	5.217	2.006	**10.461**	3.068
Exercise	5.231	1.761	6.820	2.351	5.573	1.675
Cooking Oil	7.068	3.839	5.937	3.408	7.999	3.735
Food Source	7.241	3.303	7.075	2.764	4.694	2.302
FB17	6.024	2.708	6.687	3.027	5.249	2.515
FB18	4.705	3.007	3.493	2.807	3.808	2.504
FB19	5.106	2.688	1.938	2.334	2.475	2.176
FB20	5.622	3.262	5.010	2.763	4.052	2.240
FB21	6.370	2.987	0.272	2.012	2.828	2.280
FB22	3.023	2.673	3.535	2.433	3.278	2.322
FB23	4.509	2.251	4.425	2.057	0.661	1.715
FB24	3.749	2.664	2.621	2.416	3.893	2.180

Table S4. A comparison of feature importance metrics derived from the Random Forest model. The table presents scores for Mean Decrease in Accuracy (MDA), which reflects the performance loss when a feature is permuted, and Mean Decrease in Impurity (MDI), which reflects a feature's contribution to node purity. Scores are shown for demographic, clinical, and dietary features across three train/test ratios. Bold values represent the features with the highest importance scores in each column.

Train/Test	Random Forest						eXtreme Gradient Boosting					
	Without bootstrapping			With bootstrapping			Without bootstrapping			With bootstrapping		
	Sen.	Spec.	F1	Sen.	Spec.	F1	Sen.	Spec.	F1	Sen.	Spec.	F1
LASSO												
80/20	38.46	68.75	45.45	38.46	75.00	45.45	38.46	62.50	41.67	38.46	50.00	38.46
75/25	43.75	70.00	50.00	43.75	75.00	50.00	50.00	**75.00**	**55.17**	37.50	70.00	42.86
70/30	36.84	66.67	43.75	36.84	75.00	43.75	52.63	62.50	52.63	37.50	62.50	42.86
5-fold	45.77	60.00	45.82	89.51	96.40	92.12	43.63	63.92	44.49	91.80	94.01	92.04
10-fold	33.33	68.75	38.59	89.40	**99.99**	91.78	48.81	59.98	47.41	92.02	**95.83**	**93.07**
MDA												
80/20	38.46	75.00	45.45	38.46	62.50	41.67	30.77	56.25	33.33	38.46	62.50	41.67
75/25	37.50	**85.00**	48.00	31.25	75.00	38.46	43.75	65.00	46.67	43.75	75.00	50.00
70/30	42.11	62.50	44.44	36.84	62.50	40.00	47.37	70.83	51.43	43.75	66.67	50.00
5-fold	41.54	65.92	44.45	88.72	95.21	90.99	41.76	67.69	45.62	90.73	95.30	92.09
10-fold	35.00	66.96	35.49	90.88	95.81	92.43	43.76	63.12	46.56	91.63	94.67	92.25
MDI												
80/20	38.46	81.25	47.62	38.46	68.75	43.48	38.46	62.50	41.67	38.46	62.50	41.67
75/25	**56.25**	56.25	**56.25**	50.00	55.00	48.48	**56.25**	60.00	54.55	56.25	70.00	58.06
70/30	36.84	58.33	38.89	31.58	62.50	35.29	36.84	66.67	41.18	56.25	70.83	58.06
5-fold	49.74	65.75	50.39	**92.51**	94.03	**92.45**	42.96	68.94	44.58	90.02	91.20	89.31
10-fold	38.33	65.89	39.27	90.22	93.97	91.28	46.63	59.23	44.84	**92.46**	92.29	91.15

Table S5. A comprehensive comparison of model performance (%) in predicting psoriasis severity. The table evaluates two classifiers, Random Forest (RF) and eXtreme Gradient Boosting (XGB), combined with three feature selection methods: LASSO, Mean Decrease in Accuracy (MDA), and Mean Decrease in Impurity (MDI). Each combination was tested on various train/test splits and cross-validation folds, under scenarios without ($n_{\text{train}} = 99 - 113$) and with bootstrapping ($n_{\text{train}} = 375$). Performance is reported as Sensitivity (Sen.), Specificity (Spec.), and F1-score (F1), with the highest-performing value in each block highlighted in bold.

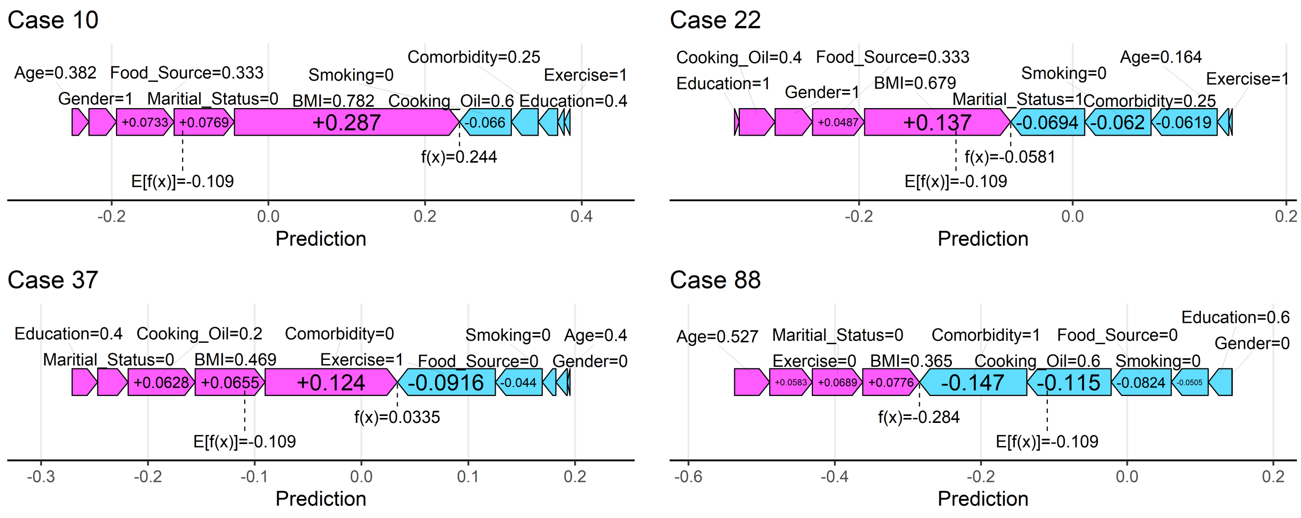

Fig. S2. Local SHAP values for four representative patients, illustrating the contribution of demographic and clinical features to the model's prediction of psoriasis severity. Positive contributions (magenta) push the prediction toward higher severity, while negative contributions (blue) push it toward lower severity. Cases 10 and 37 correspond to patients with higher predicted severity ($Y = 1$), whereas Cases 22 and 88 correspond to patients with lower predicted severity ($Y = 0$).

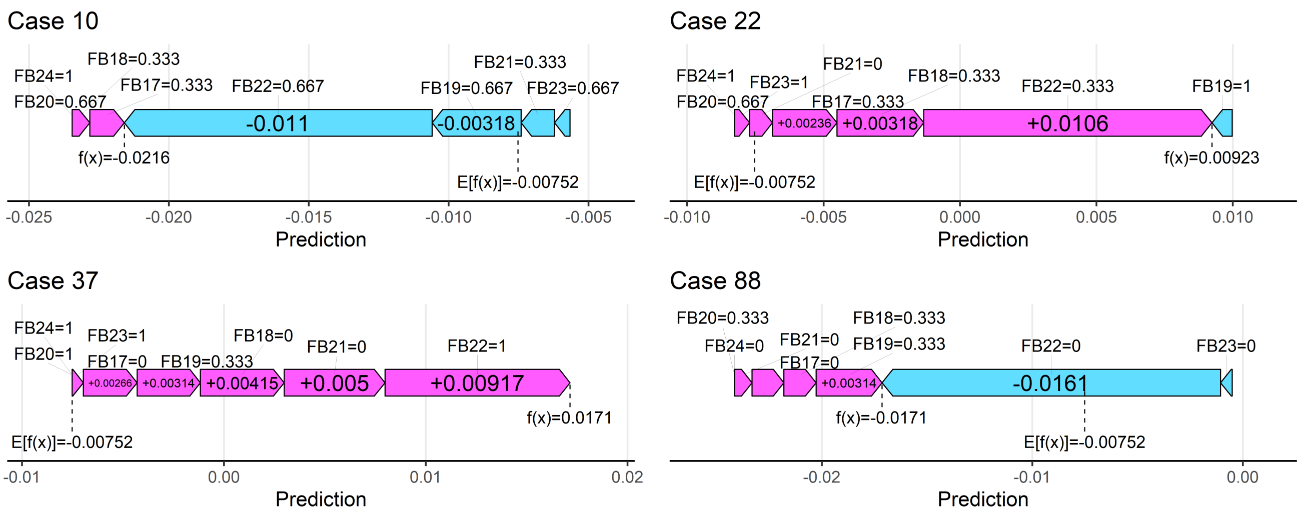

Fig. S3. Local SHAP values highlighting the contribution of individual dietary features (FB codes) to model predictions of psoriasis severity in four representative patients. Positive SHAP values (magenta) indicate dietary patterns driving predictions toward higher severity, while negative SHAP values (blue) indicate contributions toward lower severity. Cases 10 and 37 correspond to patients predicted with higher severity ($Y = 1$), whereas Cases 22 and 88 correspond to patients predicted with lower severity ($Y = 0$).
